# Quantifying Upper Limb Movement During Naturalistic Driving: A Clinically Informed Ecological Approach

**DOI:** 10.3390/s26103121

**Published:** 2026-05-15

**Authors:** Carly R. Rankin, Dwayne L. Mann, Shamsi Shekari Soleimanloo, Kalina R. Rossa, Karen A. Sullivan, Paul M. Salmon, Cassandra L. Pattinson, Simon S. Smith

**Affiliations:** 1 Faculty of Health, Medicine and Behavioural Sciences, The University of Queensland, Brisbane, QLD 4072, Australia; 2 School of Electrical Engineering and Computer Science, The University of Queensland, Brisbane, QLD 4072, Australia; d.mann@uq.edu.au; 3 School of Psychology, The University of Queensland, Brisbane, QLD 4072, Australia; s.shekarisoleimanloo@uq.edu.au; 4 Life Course Centre, The University of Queensland, Indooroopilly, QLD 4068, Australia; simon.smith@uq.edu.au; 5 Child Health Research Centre, The University of Queensland, South Brisbane, QLD 4101, Australia; k.rossa@uq.edu.au (K.R.R.); c.pattinson@uq.edu.au (C.L.P.); 6 School of Psychology and Counselling, Queensland University of Technology, Brisbane, QLD 4000, Australia; karen.sullivan@qut.edu.au; 7 The Centre for Human Factors and Systems Science, University of the Sunshine Coast, Sippy Downs, QLD 4556, Australia; psalmon@usc.edu.au

**Keywords:** wearable sensors, upper limb movement, naturalistic driving, accelerometry, convex hull volume, human motion analysis, return to driving

## Abstract

Limb movement is an important component of control during safety-critical tasks such as driving. Restricted movement, such as limitations associated with an injury or surgery to the upper limb, may impact driving safety. However, the degree of upper limb movement required for driving is not well described outside of traditional laboratory settings. There is a need for new affordable, accessible, reliable and accurate measures of normative limb movement to guide decisions about driving capacity. This feasibility study applied a volume estimation approach to wrist-worn triaxial accelerometry data to quantify upper limb movement during naturalistic driving in a young adult population. A sample of 89 participants wore accelerometers while engaging in daily driving activity over a two-week period. Results demonstrated a distribution of movement volumes, consistent with variation in individual driving behaviour. This volume estimation approach has strong potential for further development as both a research tool and clinical assessment method, particularly in rehabilitation and return-to-driving assessments following upper limb injury or surgery.

## 1. Introduction

The measurement of human upper-limb movement in real-world contexts has important implications for rehabilitation, injury recovery, and the determination of functional readiness for complex tasks such as driving. Traditional clinical assessments rely heavily on observation or laboratory-based motion capture, limiting their ecological validity and failing to capture daily functional use [1]. There is a growing need for objective and scalable methods to quantify upper limb movement in naturalistic settings, particularly during the recovery period following injury or surgery [2,3,4,5,6,7,8], and calls to develop new measurement approaches and analyses [2,9,10].

Recent studies have increasingly used wearable sensors to examine driver behaviour and activity patterns in naturalistic driving environments. Accelerometry derived from wrist-worn or body-mounted devices has been used to detect behavioural states such as fatigue, distraction, or general activity levels during driving tasks [11,12,13]. Naturalistic driving studies incorporating sensor technologies have demonstrated the value of continuous monitoring for understanding real-world driver behaviour and performance [14]. In many of these approaches, inertial signals are analysed using classification or pattern-recognition techniques to identify driver states or behaviours over extended monitoring periods [15,16]. Recent advances in wearable technology, particularly triaxial accelerometers embedded in wrist-worn devices, now provide the potential for continuous and unobtrusive monitoring of limb movement in daily life [17,18,19,20,21]. These tools provide a unique opportunity to supplement or replace conventional clinical or laboratory assessments with high-frequency, real-world data derived from naturalistic limb movement behaviours. A comparative table summarising related works and detailing key differences in research methodologies, devices, key findings and relevance to limb movement is outlined below in Table 1.

Driving is a functionally complex and biomechanically demanding task. Both gross and fine upper limb movements are required [22], and any restrictions to these movements may have consequences for driving safety [23,24]. However, there is currently very limited published data to support normative movement for the upper limbs while driving [7,25,26]. Such data is needed to quantify any changes in limb dynamics associated with limb restrictions.

Characterising the movement envelope explored by the upper limb during driving may provide complementary information to existing behavioural detection approaches, particularly in rehabilitation contexts where clinicians are interested in the extent and variability of functional limb use. In this context, convex hull volume estimation provides a potential method for summarising the three-dimensional spread of wrist acceleration trajectories during naturalistic driving.

Convex hull volume estimation provides a compact descriptor of the spatial envelope explored by a triaxial acceleration trajectory. Rather than summarising movement using point-based metrics such as mean acceleration or vector magnitude, the convex hull approach captures the full three-dimensional spread of the signal, representing the overall range and variability of motion generated during a task. This characteristic may be particularly useful for describing complex, non-repetitive activities such as driving, where upper limb movements occur across multiple planes and vary continuously over time. Although convex-hull-based volumetric metrics have been applied in biomechanics more broadly [27,28], they have not been widely used in naturalistic driving. Meanwhile, wrist-worn accelerometers are increasingly used in driving research, but typically for behaviour/state classification rather than as a movement-volume descriptor such as convex hull volume [29].

Quantitative measurement of movement during functional tasks is widely used in rehabilitation research to evaluate recovery and intervention effectiveness. For example, systematic reviews of post-stroke rehabilitation interventions commonly report quantitative gait and mobility metrics such as gait speed, step variability, and sensor-derived movement parameters as indicators of functional performance [30]. At the same time, considerable variability exists in how movement and functional outcomes are measured across rehabilitation studies. Reviews of lower-limb amputation rehabilitation literature have highlighted substantial heterogeneity in the functional and biomechanical metrics used to characterise recovery [31]. This variability reflects the complexity of measuring real-world functional movement and highlights the ongoing need to explore and refine quantitative descriptors capable of capturing meaningful aspects of human movement behaviour.

This paper describes the application of the convex hull volume estimation to three-dimensional (3D) accelerometry data to capture dynamic aspects of wrist movement during naturalistic driving. By enclosing the triaxial accelerometry trajectory in 3D space and computing the resulting volume, the method yields a single, interpretable index of how broadly and variably wrist motion unfolds under continuous, non-repetitive control demands.

Our primary goal was to characterize typical wrist movement patterns during driving. A second goal was to evaluate the feasibility of applying this method to wrist-worn accelerometry data collected during naturalistic driving. The focus is on methodological feasibility and proof of concept, rather than direct biomechanical validation or hypothesis-driven inference. As such, this work represents an early-stage development of the approach, intended to inform future validation and application studies.

## 2. Materials and Methods

### 2.1. Study Design and Participants

This analysis represents a secondary use of data collected within a larger randomized controlled trial examining sleep and naturalistic driving behaviour in young adults [32]. The original study design was not specifically intended to characterise upper limb movement patterns, and therefore detailed information regarding anthropometry, vehicle characteristics, and individual driving styles was not systematically recorded.

**Figure 1:**
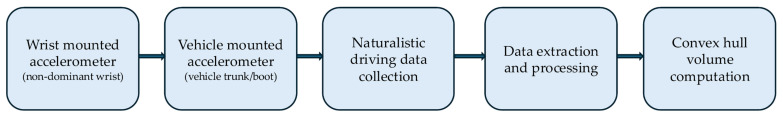
Overview of the data collection and analysis workflow. Wrist-mounted and vehicle-mounted accelerometers were used to collect naturalistic driving data. Accelerometry signals were extracted and processed to identify active driving segments, after which convex hull volumes were computed from the three-dimensional acceleration trajectories.

Baseline (pre-randomization) data from 89 participants (aged 18–24 years) were included in the current analysis. The participants completed a two-week naturalistic driving and actigraphy monitoring period and had valid wrist and vehicle sensor data available for convex hull movement analysis (overview of workflow shown in Figure 1).

All participants were required to hold a current Australian driver’s licence, have access to a personal vehicle, and report no recent history of upper limb injury. Ethics approval was obtained from the University of Queensland Human Research Ethics Committee (Approval Number: 2019/HE001269). All participants provided written informed consent prior to participation.

### 2.2. Instrumentation

Participants wore GENEActiv Original wrist-worn accelerometers (Activinsights Ltd., Kimbolton, UK) on their non-dominant wrist. These devices recorded triaxial acceleration at a sampling rate of 10 Hz and were worn continuously over the two-week assessment period. Identical accelerometers were also affixed inside the trunk (boot) of each participant’s vehicle to capture chassis motion in a standardized orientation, allowing independent assessment of both limb and vehicle-based acceleration.

### 2.3. Data Reprocessing and Convex Hull Volume Estimation

Raw accelerometry data were extracted with GENEActiv software (V3.3, Activinsights Ltd., Kimbolton, UK), and processed into a simple time series (i.e., date, time, and acceleration in X, Y, Z dimensions).

Driving periods were identified using activity thresholds applied to the vehicle-mounted accelerometer signal, allowing separation of vehicle motion associated with active driving from stationary periods. In-vehicle accelerometers were retrieved from the study vehicles at the end of each naturalistic assessment phase, and the recorded data downloaded using ActivInsights software (Version 3.3) for each participant. Individual driving episodes were identified from the vehicle accelerometer signal using threshold-based detection of vehicle motion. Specifically, discrete driving segments were defined as periods in which the absolute longitudinal acceleration signal exceeded empirically determined activity thresholds, allowing separation of vehicle motion from stationary periods. Signal envelopes were computed using a Hilbert transform approach to facilitate identification of sustained activity periods [33]. Driving segments were then evaluated based on elapsed duration and acceleration magnitude. Events exceeding longitudinal acceleration thresholds (0.35 g for acceleration and 0.45 g for deceleration) [34], and lateral acceleration thresholds (absolute value ≥ 0.5 g) [35] were retained as active driving periods, consistent with thresholds used in previous naturalistic driving research. The corresponding time segments from the wrist-mounted accelerometer were then extracted for subsequent convex hull volume analysis.

**Figure 2:**
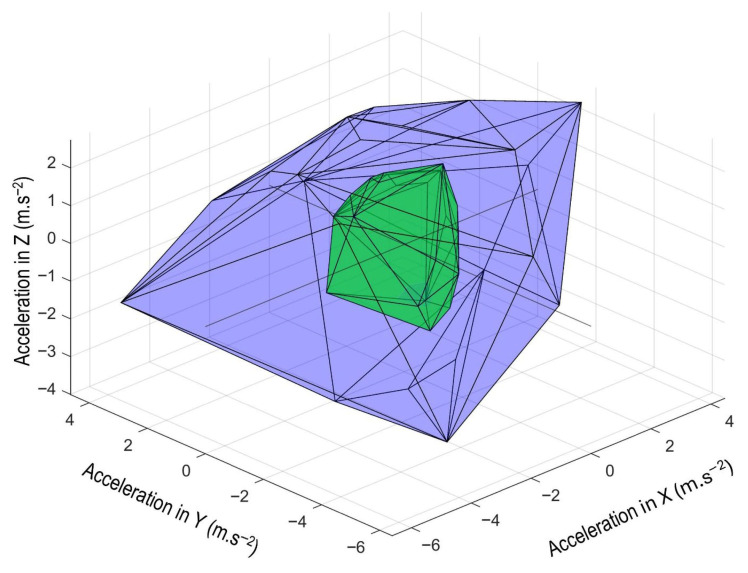
Example polyhedrons (from which convex hull volumes are then determined) for vehicle and wrist acceleration from a single drive, visualised in three-dimensions. Outer polyhedron (blue) represents limits of wrist acceleration, while inner polyhedron (green) displays limits of vehicle acceleration.

**Figure 3:**
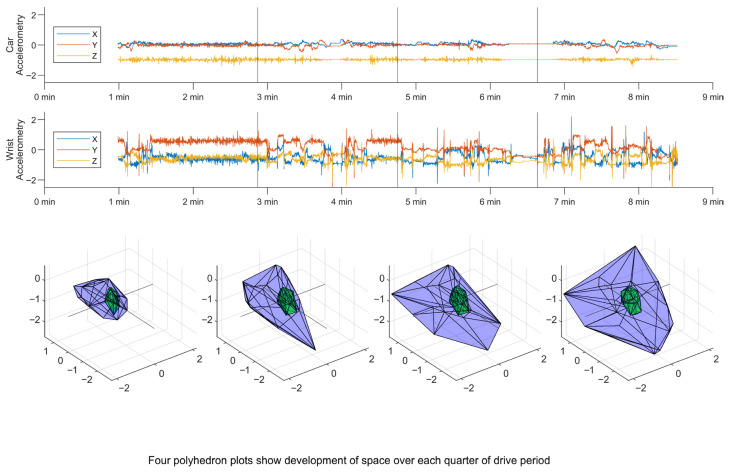
Time-series data showing how convex hull volume evolves during a drive. The car signal shows fore/aft acceleration in x, left/right in y, and up/down in z (z appears as −1 due to the sensor orientation capturing gravity). In contrast, the wrist sensor is free to move. The lower four plots show the corresponding polyhedron development over each quarter of the drive period (demarcated by faint vertical lines in top subplots), with outer polyhedron (blue) representing limits of wrist acceleration and inner polyhedron (green) displaying limits of vehicle acceleration.

For each driving period, convex hull volumes were computed from the corresponding three-dimensional acceleration data independently for both the wrist and vehicle-based devices. While several three-dimensional convex hull algorithms have been described [36,37,38], the overarching objective is that the convex hull represents the smallest polyhedron enclosing all co-ordinate data points (i.e., X, Y, and Z acceleration data in the current study, example polyhedrons for both wrist and vehicle movement shown in Figure 2). Accordingly, the convex hull captures the overall range of motion during each driving period, providing a scalar estimate of movement complexity and spatial extent (example showing evolution of hull volume across a single driving period shown in Figure 3). All convex hull vertices and their associated volume measurements were derived using the built-in ‘convhull’ function within MATLAB (R2024b, The MathWorks Inc., Natick, MA, USA). The units for the derived volume are somewhat unconventional, being the acceleration cubed (g^3^), or in physical units, m^3^·s^−6^.

### 2.4. Statistical Analysis

Descriptive statistics were calculated for each participant’s wrist-based movement volume. Quartiles, the interquartile range (IQR), and outlier thresholds (1.5 × IQR) were used to identify sessions with atypical, high-movement outliers. To reduce the possibility that wrist motion was artefactual or vehicle-induced, convex hull volumes derived from the wrist data were compared with those from the vehicle-mounted accelerometers. A constrained range (1st–99th centile) to exclude extreme outlier points, occasionally occurring in raw data, was used.

Data distributions were inspected visually (histogram, boxplot) and statistically (Shapiro–Wilk test) for normality. Given the observed right skew in wrist movement volumes, a simple log_10_ transformation was applied to explore whether normality could be improved. While the transformation reduced skewness and improved symmetry, analyses and figures are presented using raw values to retain clinically interpretable units.

## 3. Results

### 3.1. Wrist Movement Volumes

**Figure 4:**
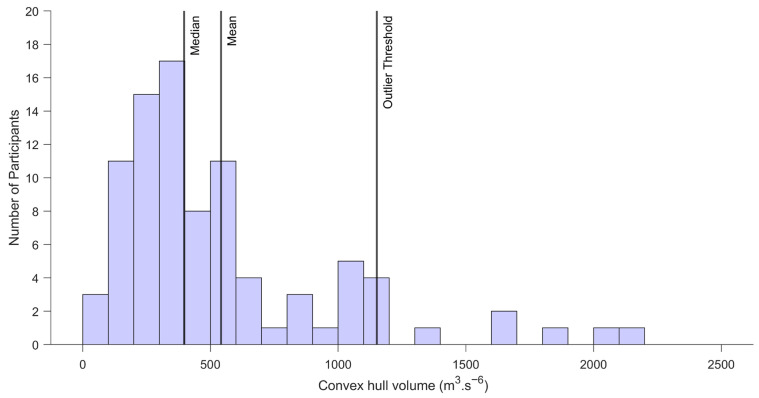
Distribution of wrist-based movement volumes during naturalistic driving. Movement volume represents the convex hull volume of the triaxial acceleration signal (g^3^), reflecting the spatial spread of wrist acceleration trajectories during driving. Most participants exhibited moderate movement volumes, with a right-skewed distribution and several high-value outliers (e.g., >1151.82 g^3^), potentially reflecting individual driving styles or upper limb compensatory patterns. A log_10_ transformation reduced skewness but was not used for reporting to preserve clinical interpretability.

Convex hull volumes derived from wrist accelerometry exhibited substantial variability across the sample. The mean wrist movement volume was 541.57 units, with a median of 396.92 units. However, values ranged from 64.72 to 2148.89 units, with an interquartile range (IQR) of 353.98. An outlier threshold, defined as 1.5 × IQR above the third quartile, was calculated at 1151.82 units. Six participants exceeded this threshold, indicating atypically high wrist movement volumes during the driving episodes. These data were used to generate a preliminary descriptive distribution of observed movement volumes within this cohort (Figure 4).

Log_10_ transformation of wrist movement volumes substantially reduced skewness (from 3.51 to –0.48) and improved distributional symmetry. However, the overall interpretation and ranking of participants remained unchanged, so results are reported in raw units for clarity and clinical relevance.

### 3.2. Vehicle Movement Volumes

During the driving periods, convex hull volumes calculated from trunk-mounted vehicle accelerometers were substantially lower and less variable than the corresponding wrist-based values. The mean volume for the vehicle accelerometers was 31.40 units, with a median of 20.54 units and a maximum of 279.99 units. The outlier threshold was calculated at 110.06 units, but few values approached this level. Summary statistics for both wrist- and vehicle-based movement volumes, including the mean, median, standard deviation, and interquartile range, are presented in Table 2.

### 3.3. Interpretation

The distribution of wrist movement volumes is consistent with interpretable inter-individual variation in upper limb use during driving. Similar volumetric convex-hull based approaches have been used to characterise movement envelopes or reachable space in robotics manipulators, where differences in volume relate to variability in kinematic capability [39] and constructs related to time- and geometrically varying convex hulls have been used to track dynamically changing motion envelopes in continuous time and motion systems [40]. In this study, six participants (6.74%) exceeded the upper outlier threshold of 1151.82 units, and the middle 50% of participants exhibited volumes between 268.48 and 620.25 units. Participants exceeding the outlier threshold may demonstrate greater voluntary motion due to ergonomic factors (i.e., vehicle design features including seating and steering configuration, and driver anthropometry), habitual movement styles, or compensatory motor strategies. In contrast, the lower and more stable vehicle-based movement volumes confirm that the observed wrist data largely reflect voluntary upper limb activity, rather than vibration or whole-body movement from the vehicle itself. The confound between the two, potentially dependent movement sources could be controlled by a subtraction approach, or by limiting interpretation to individual changes (e.g., in a n-of-1 trial paradigm).

## 4. Discussion

While prior wearable-sensor studies in driving research have largely focused on detecting driver states or behaviours, the present study demonstrates the feasibility of collecting wrist movement data during naturalistic driving using wrist-worn accelerometry, and of applying convex hull volume estimation to describe these movements. The findings suggest that convex hull analysis can describe meaningful inter-individual differences in upper limb movement patterns during driving. The values generated during on-road driving may serve as a preliminary descriptive distribution of observed movement volumes within this cohort. The observed right-skewed distribution, and identification of high-movement outliers, supports the technique’s potential to capture variability associated with ergonomic factors, motor behaviour, or compensatory strategies. However, the degree of inter-individual variability observed in this study also suggests that convex hull volume estimates cannot be used to directly define a simple threshold-based or binary “safe versus unsafe” range for driving without further validation. Unlike scalar metrics such as vector magnitude, which reflect instantaneous acceleration intensity, convex hull volume summarises the three-dimensional spread of acceleration trajectories, providing an aggregate descriptor of the spatial movement envelope explored during a driving task. This distinction highlights that convex hull volume and scalar metrics capture different characteristics of movement, with the former reflecting cumulative spatial extent and the latter reflecting instantaneous magnitude. The intention is not to suggest that convex hull volume is inherently superior to existing metrics, but rather that it offers a complementary perspective on movement behaviour.

Variability in limb movement is an expected feature of naturalistic driving, reflecting differences in vehicle ergonomics (e.g., vehicle class, seating and steering configuration), driver anthropometry, habitual movement patterns, and task strategies. This variability may also reflect differences in vehicle characteristics such as steering wheel dimensions, or driver-assist technologies. Differences in route complexity, such as a higher frequency of turns, as well as variations in driving speed or overtaking behaviour, may also contribute to differences in observed movement patterns during naturalistic driving. In this context, the primary utility of this approach lies in characterising movement patterns and monitoring change over time, particularly within individuals, rather than establishing absolute population-level cut-points. Convex hull volume should therefore currently be interpreted as a relative descriptor of overall movement variability rather than a direct proxy for joint-level kinematics or established clinical rehabilitation measures. This may be particularly useful for intra-individual comparison over time, such as tracking changes in movement behaviour during recovery or rehabilitation.

More broadly, accurate quantification of limb movement during functional activities is increasingly recognised as valuable in rehabilitation science. Interventions aimed at improving sensorimotor function, including sensorimotor training programs and proprioceptive interventions, rely on the ability to measure changes in movement behaviour and joint control during real-world tasks. Driving is a complex functional activity requiring coordinated sensorimotor control, continuous environmental monitoring, and the integration of motor and cognitive processes. Similar coordination demands are observed across many functional tasks studied in rehabilitation research, including gait and balance control. For example, vestibular and sensorimotor impairments have been shown to influence functional independence and mobility in neurological populations [41]. In addition, systematic evidence demonstrates that performing motor tasks under dual-task conditions can significantly influence motor stability and coordination [42]. These findings highlight the importance of understanding how movement patterns are expressed during complex real-world activities and support the potential value of wearable sensing approaches for capturing functional motor behaviour outside controlled laboratory environments. Recent systematic evidence also suggests that supportive devices, such as compression garments, may influence proprioceptive accuracy and joint position awareness, further highlighting the importance of objective movement monitoring in rehabilitation contexts [43]. Wearable sensing approaches that capture naturalistic movement patterns may therefore provide a valuable complement to traditional clinical assessments when evaluating functional behaviour during real-world tasks and may support future research examining recovery following upper limb injury or surgery.

Such an approach may be especially valuable for monitoring deviation from an individual’s baseline during recovery or rehabilitation, where relative change may be more informative than comparison to a fixed reference range. Exploratory log_10_ transformation of the wrist movement improved distributional symmetry. Transformation, and other scaling approaches such as z-scores, may be useful in the future development of reference ranges, depending on the distribution returned by large samples. For clinical interpretability, results were retained in raw units.

This approach may provide a scalable and interpretable measure of movement variability and spatial range, offering an alternative to traditional motion capture techniques. Unlike laboratory-based methods, this technique allows longitudinal, unobtrusive monitoring of upper limb activity with minimal participant burden, making it suitable for use in real-world clinical and functional assessments. While clinical observation and simulator-based assessments can provide robust insight into driving-related performance, they are resource-intensive and not readily scalable for repeated or longitudinal assessment, particularly during temporary post-operative recovery, highlighting the value of complementary wearable-based approaches. From a clinical perspective, this approach offers promising utility for monitoring upper limb function during meaningful tasks such as driving. For example, occupational therapists involved in post-operative rehabilitation could use convex hull metrics as an adjunct to functional assessments, particularly when evaluating readiness to return to driving. For this purpose, further normative data in relevant clinical or age-matched samples would be necessary, alongside evidence of validity and test reliability. In the longer term, this approach may support the development of population-level reference distributions describing typical wrist movement during driving. Such reference data would be expected to reflect substantial inter-individual variability and may require stratification by factors such as vehicle type, driving context, and driver characteristics. In the near term, practical application is more likely to focus on contextualising an individual’s movement patterns relative to their own baseline or emerging normative distributions, rather than applying fixed threshold values. Further work is required to establish robust reference datasets, determine sensitivity to clinically meaningful change, and evaluate how convex hull metrics relate to functional recovery and return-to-driving outcomes. Objective monitoring of real-world wrist activity over time may reveal compensatory movement patterns or incomplete recovery that may not be evident during brief in-clinic evaluations. With further validation, this approach may ultimately support more tailored and evidence-informed return-to-driving recommendations.

This study provides preliminary descriptive data that may inform the development of normative movement benchmarks under controlled conditions. From a methodological perspective, this work represents progression from early conceptualisation to experimental proof of concept (Technology Readiness Level 1–3). Future work could compare these convex hull volume estimates with established normative joint range of motion data from the allied health and biomechanics literature, providing a bridge between wearable-derived metrics and conventional clinical measures. Future research should also focus on refining the analytical parameters, validating the approach across broader populations, and correlating convex hull volumes with functional outcome measures such as grip strength, range of motion, or return-to-driving timelines.

This study has several limitations. First, although the trunk-mounted accelerometer provided an independent reference measure of vehicle motion, the study did not include a direct biomechanical ground-truth method such as optical motion capture or in-cabin video analysis to verify the specific joint movements contributing to the recorded wrist accelerometry signals. Future work incorporating such reference methods would allow more detailed validation of the relationship between convex hull volume estimates and underlying upper limb kinematics during driving tasks. Second, the study draws on a secondary analysis of community dwelling young adults, with participants not selected based on upper limb clinical criteria. As such, the findings cannot yet be generalised to surgical or injured populations. It will be important to determine if clinical limb restrictions result in discriminable differences in movement volume estimates. In addition, no detailed information was available regarding physical anthropometric attributes of participants, vehicle characteristics including in-cab ergonomic configuration, or individual driving styles and positions. Third, while convex hull volume estimation provides a novel scalar output, its correlation with validated clinical measures of function remains to be established. For example, the relationship between these estimates and clinical metrics such as joint range of motion remains to be determined. Fourth, the benefit of the convex hull estimation approach over other potential indices of movement has not been clearly demonstrated. Comparison of analytic approaches, such as sensitivity analyses, may provide insights to optimize data treatment. Lastly, the relationship between limb movements determined by this measure, and safety or performance outcomes also remains to be determined. Future methodological work may also explore the application of machine learning and artificial intelligence approaches to large-scale accelerometry datasets in order to identify complex movement patterns and relationships that may not be captured by single summary descriptors.

## 5. Conclusions

The use of discrete wrist-worn wearables offers a novel, practical method for quantifying upper limb movement during real-world driving. The analysis approach provides a unitary representation of movement variability and spatial extent. This approach was designed to support ecologically valid measurement with minimal participant burden. As demonstrated in this feasibility analysis, the method can detect inter-individual variation in movement patterns and distinguish voluntary limb use from vehicle-induced motion. Findings indicate that most participants fell within a consistent mid-range of movement volumes, with a small subset showing markedly higher values. This provides preliminary descriptive data on driving-related upper limb movement in healthy adults. Exploratory log_10_ transformation reduced skewness but did not change interpretation, supporting the use of raw units for clinical clarity. To our knowledge, this is the first study to apply convex hull volume estimation to wrist-worn accelerometry data collected during naturalistic driving. This approach may provide a foundation for future objective functional evaluation in populations recovering from upper limb injury or surgery. With further validation, this prospective method may complement traditional assessments by offering real-world insights into movement behaviour, recovery trajectories, and rehabilitation progress.

## Figures and Tables

**Table 1:** Comparative table of related works.

Study	Device	Focus/Methodology	Findings	Relevance to Limb Movement
Yang & Hsu, 2010 [11]	Wearable accelerometers	Human movement monitoring using accelerometry-based activity detection	Demonstrated widespread use of wearable sensors for real-world motion monitoring	Not specific to driving tasks
Liang & Lee, 2010 [12]	Driving simulator instrumentation	Experimental analysis of cognitive and visual distraction	Combined distraction effects on driver performance	Laboratory setting rather than naturalistic driving
Martins et al., 2021 [13]	Wearable physiological sensors	Review of wearable fatigue monitoring approaches	Demonstrated feasibility of wearable fatigue detection	Focus on physiological fatigue rather than limb kinematics
Dingus et al., 2006 [14]	Vehicle sensors and cameras	Naturalistic driving behaviour monitoring using continuous in-vehicle sensing	Demonstrated feasibility of large-scale naturalistic driving monitoring	Focused on safety events rather than limb movement
Tan et al., 2024 [15]	Driver monitoring systems and sensors	Machine learning based driver distraction recognition	Advanced detection of distraction behaviours	Focus on behavioural state detection
Meiring & Myburgh, 2015 [16]	Vehicle and wearable sensors	AI-based classification of driving style and behaviour	Identified algorithms for analysing driving behaviour	Focus on behaviour classification rather than limb motion
Present study	Wrist-worn triaxial accelerometer	Convex hull volume estimation of wrist movement	Quantifies spatial envelope of wrist acceleration during naturalistic driving	Requires further validation with biomechanical reference measures

**Table 2:** Summary statistics comparing wrist-based and vehicle-based movement volumes.

Measure	Wrist Movement Volume	Vehicle Movement Volume
Mean	541.57	31.40
Median	396.92	20.54
Standard Deviation	443.12	38.54
Min	64.72	0.48
Max	2148.89	279.99
IQR	353.98	42.01
Upper Outlier Threshold	1151.82	110.06

## Data Availability

The data presented in this study are available on reasonable request from the corresponding author. The data are not publicly available due to ethical restrictions.
